# Motor and non-motor improvements following short-term multidisciplinary day-clinic care in Parkinson´s disease

**DOI:** 10.1007/s00702-022-02562-w

**Published:** 2022-11-06

**Authors:** Patricia Krause, Sara Berking, Melanie Astalosch, Raymond Grünheid, Andrea A. Kühn

**Affiliations:** Movement Disorder and Neuromodulation Unit, Department of Neurology, Charité, University Medicine Berlin, Charité – Universitätsmedizin Berlin, Campus Mitte, Charitéplatz 1, 10117 Berlin, Germany

**Keywords:** Parkinson´s disease, Parkinson day-clinic, Multidisciplinary therapy, Quality of life

## Abstract

**Background:**

Inpatient as well as outpatient care does often not meet PD-patients’ individual needs.

**Introduction:**

Day-clinic concepts encompassing a multidisciplinary team as well as therapy adjustments accompanying everyday demands aim at filling this gap.

**Methods:**

This is a retrospective study on short-term effects of a 3 week multidisciplinary rehabilitation program in patients with Parkinson´s disease (PD) embedded in a specialized movement disorder day-clinic. We analyzed short-term outcome of motor and non-motor symptoms (NMS) in 143 PD-patients (mean age 65.3 ± 11.9 years; Hoehn-and-Yahr-score 2.6 ± 0.7) after 3 weeks with 7.4 ± 1.8 active days of interdisciplinary day-care treatment. Participants attended the day-clinic in groups of five patients at a time. Improvements were evaluated by comparison of standardized physical therapy assessments, disease specific scores for motor symptoms (MDS–UPDRS III), mood (BDI), quality of life (PDQ39, SF36), sleep (PDSS, ESS), impulsiveness (QUIP), apathy (SAS), cognition (MMST), as well as change in medication before and directly after the intervention.

**Results:**

MDS–UPDRS motor score improved significantly by 22.9 ± 21.5% (*p* < 0.001) and was accompanied by a significant reduction of imbalance, immobility, and weakness ranging between 6% and 17% in standardized physical therapy tests. In addition, all disease-specific non-motor scales improved significantly.

**Conclusions:**

A multidisciplinary day-clinic approach can support benefit on motor, non-motor symptoms and QoL in PD-patients. Given the increase in PD incidence and prevalence as well as the significant treatment effects shown here, more day-clinic treatment opportunities ought to be implemented to improve PD treatment adapted to everyday challenges while still reducing costs to the health care system.

## Introduction

Parkinson´s disease (PD) is one of the fastest growing neurological disorders in the world (Dorsey et al. 2018) and PD patients are reported to be hospitalized ~ 1.5 times more frequently than non-PD patients (Gerlach et al. 2011). Consequently, the demand for novel approaches to an all-encompassing and more personalized treatment is increasing. Treatment of PD has become more and more challenging due to the growing evidence of genetically caused Parkinsonian syndromes, different PD subtypes with several motor as well as non-motor symptoms, complex medication plans that require frequent adjustments, and the increasing use of invasive therapies, such as drug pumps and deep brain stimulation. Optimal therapy requires a multi-professional team consisting of neurologists, PD nurses, physical and speech therapists. Disease progression often calls for the involvement of nutritional advisors, social service, occupational therapists and the cooperation with other medical departments including psychiatrists and psychologists.

While inpatient treatment offers interdisciplinary treatment options and observation times over circadian rhythms, its artificial environment does not allow for treatment adjustments that reflect patients’ everyday challenges. Outpatient care offers more frequent (follow-up) appointments but often lacks a multidisciplinary approach and has major time constrains during the direct interaction with the doctor. Furthermore, it requires a high degree of autonomy from an older patient cohort with a particular need for more complex treatment adjustments. Consequently, contemplated new regimen are often not realized at follow-up.

Up to now, these standard medical therapies are not only expensive and time-consuming, but also lack an individualized and patient-empowering approach (Buhmann et al. 2016; Oguh and Videnovic 2012). Day-clinics embedded in the infrastructure of specialized hospitals offer a comprehensive interdisciplinary alternative for PD patients (Fründt et al. 2018). They combine the professional resources of inpatient care, while at the same time, they offer regular outpatient treatment evaluation in patients’ real-life environment (Fründt et al. 2018; Titova and Chaudhuri 2017). Hence, this new type of PD care provides a cost effective and yet highly individualized treatment alternative for a growing number of patients with neurodegenerative diseases.

Here, we present the results of a retrospective study that investigates the short-term effects of a multidisciplinary rehabilitation program in > 140 patients with Parkinson’s disease embedded in a movement disorder day-clinic for 3 weeks. The purpose of this study was to investigate the benefits of day-clinic care in PD with regard to motor as well as non-motor symptoms including quality of life and mood.

## Methods

### Treatment concept, scores and questionnaires

During a 3-week treatment period, two groups of PD-patients attended either nine (3 days/week group, 3G) or six (2 days/week group, 2G) whole days of day-clinic therapy every second day. An alternation between a treatment day and a day off provides a basis for immediate real-life testing of therapeutic approaches and the opportunity to adapt as needed on the following day. Each group comprised five patients and each treatment day lasted approximately 6–7 h.

On the day of admission, medical history, neurological examination with emphasis on motor as well as non-motor symptoms, DBS settings, if applicable, and clinical scores (MDS–United Parkinson´s Disease Rating Scale I–III) were taken by the physician. Dopaminergic medication was converted into levodopa equivalence daily dosages (LEDD). Patients further completed disease-specific rating scales with focus on sleep (PDSS and ESS), mood (BDI-II), quality of life (PDQ39, SF36), psychosocial functioning (SCOPA-PS), impulsiveness (QUIP) and apathy (Starkstein Apathy Score) with support of a PD nurse if necessary (Beck et al. 1961; Marinus et al. 2003; Peto et al. 1995; Starkstein et al. 1992; Trenkwalder et al. 2011; Ware and Sherbourne 1992; Weintraub et al. 2009). Furthermore, physical therapists examined PD-specific impairments with respect to balance, mobility and strength by means of Berg-Balance-, Functional Reach-, Timed-Up-and-Go-Test as well as Sit-to-Stand-Test (Berg et al. 1992; Bohannon 1995; Podsiadlo and Richardson 1991; Weiner et al. 1992). An interdisciplinary conference with the involved team members of the day-clinic takes place to discuss the patients’ individual needs at the beginning of the treatment period; this serves as the foundation for patient individualized treatment plans. This baseline evaluation also serves as the reference to determine treatment success at the end of the treatment period. In this pretest–post-test design, all examinations, scales and scores on admission (baseline) were repeated at discharge following 3 weeks of treatment.

Routine day-clinic treatment included daily consultations with a movement disorder specialist for therapy adjustments as well as one to two sessions of relaxation sessions/relaxation therapy, individualized and group physio- as well as speech-therapy sessions and two Tai-Chi courses per week. A neuropsychological intervention was performed by a psychologist and the treating neurologist with a focus on disease understanding, acceptance and management. Adjustment of treatment plan was performed individually for each patient including increase of frequency of physiotherapy and/or speech therapy based on clinical tests and personal needs compiled on admission day. Furthermore, patients were offered nutritional advice, social services and one-on-one psychological interviews, if needed. Because of the day-clinic’s affiliation to the University hospital, extended interdisciplinary diagnostic was available if needed.

### Patients

Between August 2018 and March 2020, 269 patients with movement disorders including 207 patients with Parkinson´s disease underwent treatment in the Charité movement disorders day-clinic. Patients were referred from either their practicing neurologist or the local outpatient clinic. PD-patients that received a short-term treatment of < 5 days and patients that had already taken part in the day-clinic treatment previously were excluded (*n* = 35 and *n* = 29, respectively). A total of 143 patients were included in the final analysis.

### Data analysis and statistics

Statistical analyses were performed using the IBM SPSS software version 19.0. Patients´ baseline and disease specific characteristics including length of stay and L-Dopa dosages were calculated by use of means, standard deviation and frequency distribution. Pre- and post-intervention scores and questionnaires were analyzed by comparison of mean admission and discharge scores, except for SF36. SF36 scales were calculated after scoring instructions previously described by Ware et al. (Ware and Sherbourne 1992). Motor benefits measured by mean UPDRS III scores were compared between patients with LEDD increase (*n* = 79) and those without or LEDD reduction (*n* = 50) after the day-clinic intervention. All scores were first tested for normal distribution using Shapiro Wilk test. Afterward, mean scores at admission and at discharge were compared using parametric *t* tests or non-parametric Wilcoxon tests. *P* value < 0.05 was considered significant. Spearman’s correlation was used to investigate the relation between motor outcome, changes in QoL and non-motor symptoms as well as LEDD-change. *P* values were adjusted for multiple comparison using stepwise comparison method.

### Ethics

Treatment evaluation was carried out as part of the clinical routine with the objective of internal success monitoring and quality control of the new clinical concept in our department for neurology. The presented analysis is not part of a clinical study. All patients signed a regular treatment contract with the department for neurology and gave informed consent for data collection. The authors confirm that the approval of our institutional review board was not required for this work. The study has been performed in accordance with the ethical standards laid down in the 1964 Declaration of Helsinki and its later amendments. We confirm that we have read the Journal’s position on issues involved in ethical publication and affirm that this work is consistent with those guidelines.

## Results

### Patients

Patients (94 male, 49 female) had a mean age of 65.3 ± 11.9 years (range 28–91 years), a disease duration of 10.8 ± 7.9 years (range 1–52) and presented with a mean Hoehn-and-Yahr-stage of 2.6 ± 0.7 (range 1–4). Twenty patients suffered from tremor-dominant, 71 patients from akinetic-rigid and 52 patients from equivalence type. Fifty-nine patients were treated with deep brain stimulation (targets: 58 Ncl. subthalamicus and 2 Gl. pallidus). With a mean attendance time of 7.42 ± 1.8 days (range 5–16 days), 70 patients attended the 3G (9 appointments) and 73 patients attended the 2G (6 appointments) (Table 1).

**Table 1:** Demographic patients´ characteristics including gender, age, Hoehn-and-Yahr-stage, disease duration, PD subtype and individuals with deep brain stimulation (DBS) and respective target structures

Patients´ characteristics (*n* = 143)	Results (mean ± SD)
Gender	49 female, 94 male
Age (years)	65.34 ± 11.9
Hoehn-and-Yahr-Stage	2.55 ± 0.7
Disease duration (years)	10.8 ± 7.9
*PD subtype*	
Tremor-dominant	20 (14%)
Akinetic-rigid	71 (49.6%)
Equivalent	52 (36.4%)
DBS (number of patients)	59
STN	58
GPi	1

Abbreviations: *PD *Parkinson´s disease, *STN *nucleus subthalamicus, *GPi *Globus pallidus internus

### Motor symptoms

Motor impairment reflected by the MDS–UPDRS III improved significantly by a mean of 23% from 32.2 ± 11.9 points to 24.7 ± 11.3 points (*p* < 0.001). Additional motor evaluation by standardized physiotherapeutic assessment demonstrated a significant 5.7 ± 11.0% reduction of imbalance in the Berg Balance Test (*p* < 0.001). Likewise, Functional Reach Test ameliorated by 16.6 ± 50.1% for the right-hand side and 10.7 ± 45.8% for the left-hand side (*p* = 0.003 and 0.019, respectively) after 3 weeks of treatment. With regard to strength and mobility, the Sit-to-Stand-Test demonstrated higher mobility after day-clinic treatment with 20.6 ± 63.5% more repetitions per minute (*p* < 0.001). The Timed-up-and-Go-Test also improved significantly with a 7.9 ± 22.5% reduction of time needed for completion of a standardized walking distance at discharge (*p* < 0.001; for details please, see Table 2A).

**Table 2:** Short-term motor outcome comparing specific scores before and immediately after day-clinic treatment measured by the overall PD motor score and additional standardized physiotherapeutic assessments presented significant improvements in the PD-specific motor impairment by 23% in the MDS–UPDRS III

Motor symptoms	Motor score	Admission (mean value ± SD) [min–max]	Discharge (mean value ± SD) [min–max]	Significance (two tailed)
(**A**)				
Severity	MDS–UPDRS III	*n* = 135 32.17 ± 11.88 [11–65]	*n* = 130 24.67 ± 11.34 [6–55]	*n* = 129 *p* < 0.001
Static balance, risk of falling	BBS	*n* = 135 48.04 ± 7.87 [6–56]	*n* = 123 50.59 ± 6.16 [29–56]	*n* = 122 *p* < 0.001
Dynamic balance	FRT_right	*n* = 130 27.67 ± 9.26 [7–60]	*n* = 121 29.66 ± 8.47 [11–63]	*n* = 117 *p* = 0.003*
Dynamic balance	FRT_left	*n* = 131 27.48 ± 9.28 [0–50]	*n* = 123 28.99 ± 9.59 [0–65]	*n* = 119 *p* = 0.019
Mobility	TUAG	*n* = 136 11.39 ± 6.13 [4–48]	*n* = 123 10.04 ± 4.78 [4–32]	*n* = 122 *p* < 0.001
Mobility, physical fitness	MSST	*n* = 133 18.37 ± 6.84 [0–39]	*n* = 120 20.86 ± 8.28 [0–50]	*n* = 118 *p* < 0.001
(**B**)				
Mood/depression	BDI-II	*n* = 133 12.26 ± 7.88 [0–44]	*n* = 131 9.18 ± 7.25 [0–37]	*n* = 131 *p* < 0.001
Impulse control disorder	QUIP	*n* = 131 3.77 ± 9.88 [0–87]	*n* = 129 2.26 ± 4.97 [0–30]	*n* = 129 *p* = 0.006
QoL	PDQ39	*n* = 129 28.50 ± 15.23 [.78–66.67]	*n* = 129 24.44 ± 15.26 [0–59.11]	*n* = 125 *p* < *0.001*
Apathy	SAS	*n* = 132 16.21 ± 6.29 [3–50]	*n* = 130 14.76 ± 5.48 [4–32]	*n* = 130 *p* = 0.006*
Sleep	ESS	*n* = 131 9.44 ± 4.90 [0–24]	*n* = 129 8.26 ± 4.80 [0–22]	*n* = 129 *p* = 0.001*
Sleep	PDSS	*n* = 133 18.45 ± 10.27 [0–59]	*n* = 129 16.24 ± 9.45 [0–43]	*n* = 129 *p* = 0.005
Psychosocial functioning	SCOPA-PS	*n* = 133 9.17 ± 5.36 [0–27]	*n* = 129 7.87 ± 8.59 [0–89]	*n* = 129 *p* < 0.001
Cognition	MMST	*n* = 130 28.66 ± 2.11 [14–30]	*n* = 128 28.91 ± 1.92 [15–30]	*n* = 127 *p* = 0.122

Additional objective motor evaluation by standardized physiotherapeutic assessments demonstrated a significant 5.7 ± 11.0% reduction of imbalance in the Berg Balance Test as well as 16.6 ± 50.1% and 10.7 ± 45.8% amelioration in the Functional Reach Test, respectively. Mobility improved by 20.6 ± 63.5% measured by the Sit-to-Stand-Test and 7.9 ± 22.5% measured by the Timed-up-and-Go-Test. B) Short-term non-motor outcome comparing specific NM-PD-scores before and immediately after day-clinic treatment presenting significant improvements in all non-motor aspects except for cognition that remained stable. Evaluation encompassed the following non-motor symptoms: Mood/depression, impulsivity/compulsivity, apathy, sleep, psychosocial and cognition evaluation by means of the respective standardized scores. Wilcoxon test used, except for * paired *t* test. Abbreviations: BBS = Berg Balance Test; FRT = Functional Reach Test; MSST = Minute-Sit-to-Stand-Test; TUAG = Timed-up-and-Go-Test; NM-PD = non-motor Parkinsons´s disease; BDI = Beck Depression Index; QUIP = Questionnaire for impulsive–compulsive Disorders; PDQ39 = Parkinson´s Disease Questionnaire; QoL = Quality of Life; SAS = Starkstein Apathy Score; ESS = Epworth Sleepiness Scale and PDSS = Parkinson´s Disease Sleep Scale; SCOPAS = Short Psychosocial Questionnaire for Patients with Parkinson’s Disease; MMST = Mini Mental State; SD = standard deviation

Patients with an increase of LEDD at the time of discharge (*n* = 79) showed a reduction of -7.4 ± 6.3 points in the UPDRS III, while patients without change in LEDD or LEDD reduction (*n* = 50) presented an UPDRS III score reduction of  – 6.2 ± 6.7 points. Both groups did not differ significantly.

### Non-motor symptoms

All examined non-motor symptoms improved significantly from baseline to end of treatment: sleep improved by 1.2 ± 3.9 (*p* = 0.001) and 2.1 ± 8.1 points (*p* = 0.005) measured by ESS and PDSS, respectively. Depressive symptoms reduced by 3.0 ± 6.2 points (*p* < 0.001). Impulsivity was reduced by 1.0 ± 4.5 points (*p* = 0.006), apathy improved by 1.5 ± 6.0 points (*p* = 0.006) and psychosocial functioning had increased by 1.3 ± 8.1 points (*p* < 0.001). QoL rated by PDQ39 had improved by 3.9 ± 8.9 points after 3 weeks of inpatient treatment (for details, see Table 2B). With regard to the SF36, the subscores ´physical functioning´, ´vitality´ and ´social functioning´ improved significantly by 3.6 ± 17.4 (*p* = 0.012), 7.7 ± 14.6 (*p* < 0.001) and 5.0 ± 24.0 points (*p* = 0.006), respectively. Cognition measured by MMST at admission compared to time of discharge did not differ significantly.

Group distribution between patients with three treatment days (3G) and two treatment days (2G) per week was nearly even with 49% versus 51% of the 143 patients, respectively. Subgroup analysis revealed no significant differences between the two groups with regard to motor and non-motor benefits. Likewise, within group analysis revealed no gender differences of day-clinic treatment benefits nor with respect to DBS treatment.

### Correlation of improvement in clinical scores and its association with demographic data

No correlation of motor or non-motor improvement was found with demographic data, such as age, disease duration, disease severity (HY stage, UPDRS III), and LEDD-change. Improvement in BDI correlated significantly with improvements of impulsivity (*p* < 0.0001; *r*_*s*_ = 0.372), sleep (*p* < 0.02; *r*_*s*_ = 0.205 in the PDSS and *p* < 0.004; *r*_*s*_ = 0.256 in the ESS), apathy (*p* < 0.001; *r*_*s*_ = 0.278), QoL (*p* < 0.0001; *r*_*s*_ = 0.376 in the PDQ39 and *p* = 0.006; *r*_*s*_ =  – 0.257 in the SF36 physical component score [PCS]) and psychosocial functioning (*p* < 0.001, *r*_*s*_ = 0.459).

### Medication

Treatment adjustment included changes in medication leading to a significant increase of the patients´ mean daily levodopa equivalent dosage of about 110 mg from referral with 669.0 ± 452.9 mg to discharge with 781.6 ± 427.4 mg L-Dopa/day (*p* < 0.001) and adjustment of DBS parameter settings.

## Discussion

Here we present the first results of a 3-week day-clinic multidisciplinary treatment in 143 advanced PD patients with and without DBS as an alternative to standard in- and/or outpatient care in our movement disorders clinic. After 3 weeks of treatment, MDS–UPDRS motor symptoms had improved by a mean of 8 points, which has to be considered clinically important. This mean motor improvement of 23% was mirrored by significant improvement in the independently established physiotherapeutic assessments for balance and mobility. Moreover, BDI improved on average by 3 points and this improvement was correlated with all further non-motor assessment including QoL. Comparison of the two different treatment groups (3G versus 2G) with 9 versus 6 treatment days, respectively, revealed no differences in treatment effects across all assessed scores and questionnaires. Moreover, analysis of differences in Hoehn-and-Yahr-Stage, initial motor score and presence/absence of DBS revealed no significant difference between treatment groups (3G versus 2G) pointing to a similar treatment benefit in a broad population of PD patients. Indeed, our patients represented a broad patient population of middle to advanced PD with a mean Hoehn-and-Yahr-stage of 2.6 and a mean disease duration of 11 years with a special focus on DBS (41%).

### Improvements of motor and non-motor symptoms as well as QoL

Keeping in mind the short-term follow-up in our cohort, the interdisciplinary day-clinic approach reached functionally meaningful and statistically significant improvements in motor as well as non-motor scales in our patients. A change of 5 points in the MDS–UPDRS motor score is considered clinically meaningful in PD (Shulman et al. 2010) and was even surpassed to 8 points in our cohort. Furthermore, it exceeds motor improvements achieved by predominantly one-dimensional approaches in prominent drug-studies in advanced PD patients as well as those reached in the best medical treatment control group in the EARLYSTIM-Trial (Barone et al. 2009; Rascol et al. 2005; Schuepbach et al. 2013). Analysis of physiotherapeutic short-term interventions such as Tai Chi and Qigong mind–body exercises demonstrated an average of 3.7 points reduction (range: 1.5–6.4) in the absolute UPDRS motor scale in randomized trials (Song et al. 2017). These were in line with several other reports on mere physiotherapeutic treatment studies (Tomlinson et al. 2014). PD-specific supervised one-to-one training and other multidisciplinary interventions, however, reached comparable motor effects to those reported in our cohort (Fründt et al. 2018; Ebersbach et al. 2010). In line with this, we do not consider our day-clinic-related treatment effects to derive from either mere medical adjustment or from physical activation alone, but rather the result of an adjustment of both imbedded in a disease-specific multidimensional intervention.

Beyond mobility, non-motor symptoms are highly relevant for QoL in PD patients (Martinez-Martin et al. 2011) and prevail in ~ 98.6% of all PD patients according to the Priamo Study (Barone et al. 2009). Only recently, the PDQ39 score is used as a primary outcome measure for clinical trials in PD. While different physiotherapy techniques yield rather heterogeneous QoL effects in PD (Tomlinson et al. 2014), several drug studies found short-term PDQ39 summary index reductions between 1.5 and 2.2 and 3.3 points, respectively (Gray et al. 2022; Barone et al. 2010). First attempts to offer a multidisciplinary rehabilitation program for PD patients in a day-care unit in a district hospital obtained a ~ 4% improvement in health-related QoL after a 6-week treatment period (Trend et al. 2002). Longer invasive neurostimulation studies report on improvements ranging between, e.g., 4.6 and 7.8 points (Schuepbach et al. 2013; Tödt et al. 2022). The ~ 9% improvement in the PDQ39 summary index reported in our cohort is in line with results reported in a previous short-term neurological day clinic (Fründt et al. 2018) as well as the 24-month results of the aforementioned STN–DBS trial (Schuepbach et al. 2013). Interestingly, the PDQ39 improvement reported here exceeds those reported following other one-dimensional approaches (Schuepbach et al. 2013; Tomlinson et al. 2014; Gray et al. 2022; Tödt et al. 2022).

**Fig. 1:**
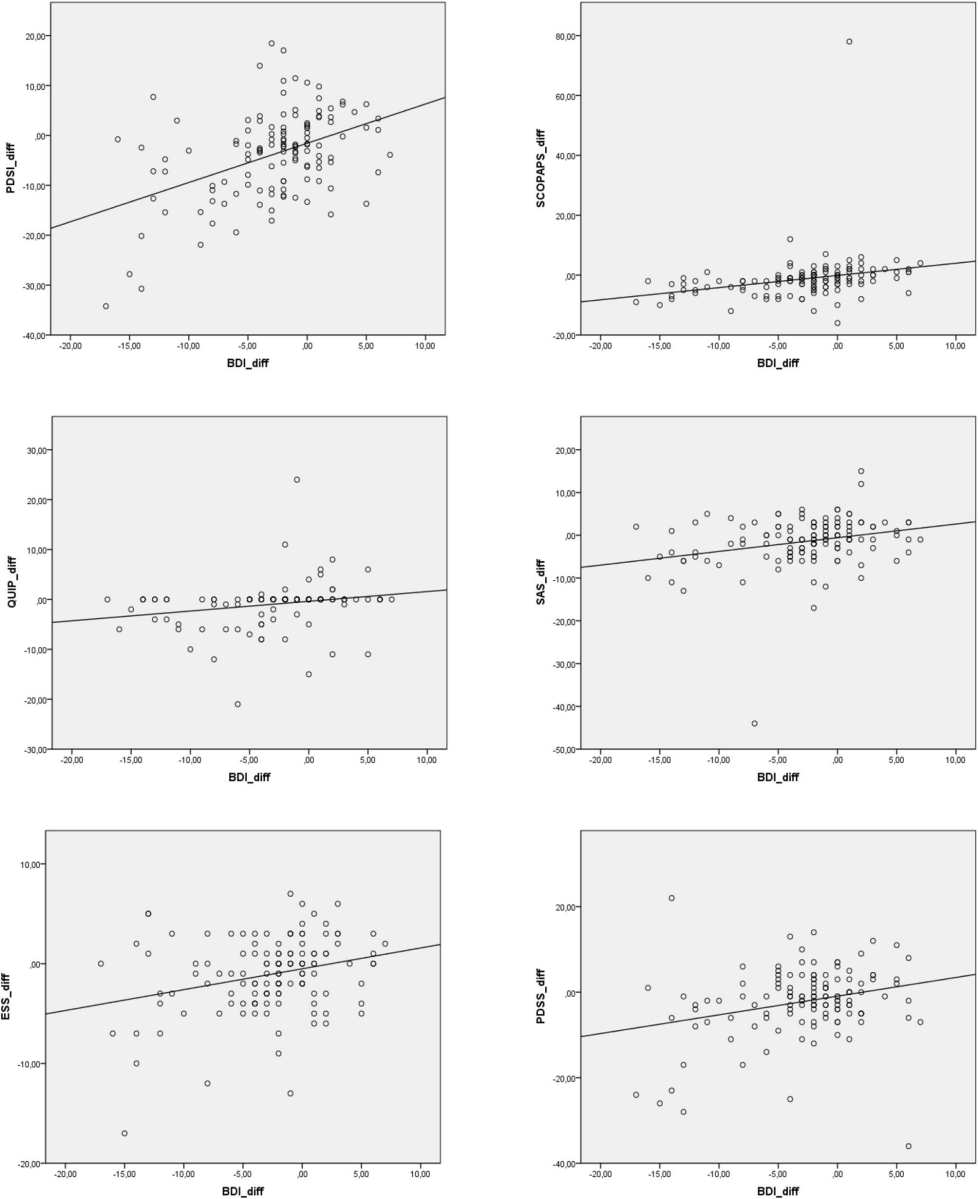
**A–F** Graphical presentation of improvements in depression scores (BDI) correlating significantly with improvements of the SCOPAS measuring psychosocial functioning (*p* < 0.01, *r*_*s*_ = .471), the PDQ39 measuring QoL in PD (*p* < 0.01; *r*_*s*_ = .390), the QUIP measuring impulsivity (*p* < 0.01; *r*_*s*_ = .364), the SAS measuring apathy (*p* = 0.01; *r*_*s*_ = .290) as well as the ESS and PDSS measuring sleep (*p* < 0.02; *r*_*s*_ = .208 in the PDSS and *p* < 0.004; *r*_*s*_ = .272 in the ESS). Abbreviations *SCOPAS* = Short Psychosocial Questionnaire for Patients with Parkinson’s Disease, *PDQ39*  Parkinson´s Disease Questionnaire, *QoL* = Quality of Life, *QUIP* Questionnaire for impulsive–compulsive Disorders, *SAS* Starkstein Apathy Score, *ESS* Epworth Sleepiness Scale and *PDSS* Parkinson´s Disease Sleep Scale, *r*_*s*_Spearman´s Rho, *diff* absolute difference

Importantly, NMS in psychiatric domains, such as depression, anxiety and apathy, is reported to be the major predictor of poor QoL (Barone et al. 2009; Rahman et al. 2008). Taken together, all NMS in our cohort had improved significantly after completion of the program (see Table 2B). Notably, the ~ 17% reduction in the BDI did not correlate with MDS–UPDRS III benefits indicating that the reduced depression score did not result merely from motor improvements. More interestingly and in line with the necessity of a multidisciplinary therapeutic approach, BDI improvements correlated significantly with improvements in the scores exploring psychosocial functioning (*r*_*s*_= 0.459), quality of life (PDQ: *r*_*s*_ = 0.376; SF36 PCS: *r*_*s*_ = 0.257), impulsiveness (*r*_*s*_ = 0.372), apathy (*r*_*s*_ = 0.278) and sleep (ESS: *r*_*s*_ = 0.256; PDSS: *r*_*s*_ = 205) (see Fig. 1). The vast majority (92%) of all patients in our day-clinic participated in a psychoeducational treatment under guidance of a licensed psychotherapist who also offered individual meetings in case of higher demand. It is conceivable that the psychotherapist sessions offered in our day-clinic had an additive effect on the non-motor improvements observed in our cohort; this warrants further exploration in an additional analysis.

### Patients´ experiences and socioeconomic implications

With regard to non-motor improvements, individual patient feedback repeatedly emphasized the direct beneficial effect patients experienced during day-clinic treatment resulting from an increase in patient contact with other patients with the same illness. Among each other, patients could relate to disease specific questions, share common burdens and worries, and the group as a whole served as a self-supportive system. Furthermore, PD specific treatment optimization takes place in parallel with patients´ usual daily challenges in their everyday environment. Especially younger patients can still attend work and are not obligated to be signed off sick for 3 weeks as would be the case in an inpatient clinic. Furthermore, this approach is cost effective as expensive hospital overnight stays are not necessary. Inpatient care is rather passive in nature since every aspect of the daily structure from bedtimes, mealtimes, medication intake and exercise is predominantly dictated by the clinical routine/externally. In contrast, the regular evaluation of patient-individualized treatment success or failure during the 3-week treatment phase together with relatives at home, at the working space, and with PD professionals in the day clinic raises patients´ awareness, conveys disease specific knowledge, and empowers the patients to adopt an active attitude toward their own PD treatment.

## Limitations, Strengths and Conclusion

A limitation of the study is its uncontrolled nature, but given a chronic progressive disease-like PD, improvements without an intervention are not to be expected. Furthermore, some of the assessments were of a self-report nature. However, motor ratings, physiotherapy assessments and validated PD-specific questionnaires were done by different trained specialists (physicians, therapists, PD nurse) each generating equal and complemental benefits. All patients were admitted from neurologists or our own outpatient clinic, so that an insufficient therapeutic concept in advance can be ruled out as a sole explanation for the improvements in our day clinic. Even more so, one can argue that although our patients had already received outpatient physiotherapy, speech therapy and regular medical advice, an additional patient-centered day-clinic approach was still able to reach significant further improvements. With regard to the novel treatment approach delivered by a new and highly motivated team confounding motivational or placebo effects leading to treatment benefits have to be considered. Improvement was measured at the time of discharge and future studies should also include long-term follow-up assessments to verify long-lasting treatment effects.

In conclusion, based on these results short-term day-clinic treatment concepts should serve as an important alternative to current traditional PD treatment. In our opinion, this multidisciplinary and comprehensive approach is an essential additive for PD treatment strategies: a cost-effective, more patient-centered and state-of-the-art infrastructure for ambulatory, non-demented PD patients.

## Data Availability

The data sets generated during and/or analyzed during the current study are available from the corresponding author on reasonable request.
